# The Interplay Between Stress and Eating Attitudes: A Cross-Sectional Study Among Romanian Physical Therapy Students

**DOI:** 10.3390/jcm14051755

**Published:** 2025-03-05

**Authors:** Corina Sporea, Maria-Veronica Morcov, Claudiu Ionut Vasile, Ioana Elena Cioca, Oana Alina Apostol, Andrada Mirea, Antoaneta Punga

**Affiliations:** 1 Faculty of Midwifery and Nursing, University of Medicine and Pharmacy “Carol Davila”, 37 Dionisie Lupu Street, 020021 Bucharest, Romania; corina.sporea@gmail.com (C.S.); andrada.mirea@umfcd.ro (A.M.); 2 National Teaching Center for Children’s Neurorehabilitation “Dr. Nicolae Robanescu”, 44 Dumitru Minca Street, 041408 Bucharest, Romania; apostol.oana.alina@gmail.com; 3 Clinical Medical Department, Faculty of Medicine and Pharmacy, “Dunărea de Jos” University of Galati, 35 A.I. Cuza Street, 800008 Galati, Romania; ionut.vasile@ugal.ro; 4 Department of Psychiatry, “Elisabeta Doamna” Psychiatric Hospital of Galati, 290 Traian Street, 800179 Galati, Romania; 5 Faculty of Medicine, University of Medicine and Pharmacy “Carol Davila”, 37 Dionisie Lupu Street, 020021 Bucharest, Romania; antoaneta.punga@umfcd.ro

**Keywords:** perceived stress, eating attitudes, quality of life, emotional exhaustion, BMI, physical therapy students, maladaptive behaviors

## Abstract

**Background:** University students often experience significant stress, which may contribute to disordered eating behaviors. **Objectives:** This study examines the relationship between perceived stress and eating attitudes among physical therapy students in Romania, exploring the impact on overall well-being and identifying predictors of maladaptive eating patterns. **Methods:** A cross-sectional survey was conducted with 192 students from the Faculty of Midwifery and Nursing, “Carol Davila” University of Medicine and Pharmacy. Two validated instruments were used: the Eating Attitudes Test (EAT-26) to assess eating behaviors and the Perceived Stress Scale (PSS-14) to measure stress levels. Statistical analyses included correlations, ANOVA, and regression models, with a significance threshold of *p* < 0.05. **Results:** Among respondents, 15.1% displayed disordered eating behaviors, with stress significantly correlating with EAT-26 subscales. Stress levels were predominantly moderate to high, with younger students reporting higher perceived stress. A significant positive correlation was found between perceived stress and disordered eating behaviors, particularly dieting and bulimia/food preoccupation. Female students reported higher stress levels than males; however, no significant gender differences were observed in disordered eating patterns. No significant associations were found between BMI, weight fluctuations, and EAT-26 scores, suggesting other factors may better explain disordered eating behaviors. Logistic regression identified perceived stress as a significant predictor of disordered eating risk, reinforcing its role in shaping maladaptive eating patterns. **Conclusions:** Stress significantly impacts eating attitudes, highlighting the need for targeted interventions to reduce stress and promote healthy coping mechanisms. Future research should investigate longitudinal patterns and the effectiveness of interventions aimed at improving student well-being. The limited sample size and the absence of sensitivity analyses are notable limitations that constrain the generalizability of the findings.

## 1. Introduction

The university years are often characterized by intense personal and academic development, but they also represent a period of significant psychological and emotional strain. Students frequently face a combination of academic pressures, financial challenges, social expectations, and uncertainty about their future, all of which contribute to heightened levels of stress. This stress is further exacerbated by the need to adapt to new environments and responsibilities, making university life particularly demanding [1,2,3,4]. Prolonged exposure to stress during this critical developmental stage can have profound effects on both mental and physical well-being, influencing various aspects of students’ lives, including their eating behaviors and energy levels [3,4,5].

While previous research has explored the relationship between stress and eating behaviors, studies specifically focusing on physical therapy students remain scarce. This population is uniquely positioned at the intersection of healthcare knowledge and personal health practices, making them a compelling group for studying how academic and professional pressures influence eating attitudes and stress.

Our study provides a novel contribution by examining this relationship within a specialized academic field where students are expected to uphold high standards of health behavior while simultaneously facing demanding coursework and practical training. Unlike previous research that often generalizes findings across broader student populations, our study focuses on a distinct subgroup that may have different vulnerabilities and coping mechanisms. By investigating perceived stress, BMI, weight perception, and disordered eating behaviors, we provide new insights into the specific challenges faced by future healthcare professionals.

Moreover, our findings have important implications for early intervention strategies aimed at preventing maladaptive eating behaviors in students who are training for professions where physical health and well-being are fundamental. Understanding these patterns allows for the development of targeted stress management and nutritional education programs that can benefit both students and, ultimately, the patients they will care for in their future careers. This research contributes to the field of eating disorders by expanding knowledge on how stress and weight-related perceptions influence eating behaviors in a specific and underexplored academic group.

The connection between stress and eating behaviors involves multiple psychological and physiological pathways. Chronic stress can trigger dysregulation of the hypothalamic–pituitary–adrenal (HPA) axis, leading to increased cortisol levels, which may influence appetite and eating habits [6]. Furthermore, emotional eating is commonly used as a coping strategy to manage stress, reinforcing maladaptive dietary patterns. Additionally, stress significantly impacts students’ quality of life (QoL) by affecting their mental health [7,8,9,10], academic performance [10,11,12,13], and social interactions [14,15]. University students often experience high levels of stress due to academic workload, financial concerns, and career uncertainty [16,17,18]. Given that QoL is a well-established determinant of mental and physical well-being, our study highlights the role of stress in shaping both eating attitudes and broader health outcomes.

Physical therapy students are particularly relevant to this research due to their exposure to health-related education, which may shape their attitudes toward body image and nutrition. Additionally, their rigorous academic schedule, including both theoretical and practical components, may heighten stress levels, making them an essential group for studying the relationship between stress and eating behaviors.

Stress is defined as the body’s response to physical or psychological stimuli, termed stressors, that disrupt homeostasis [19]. This response involves a complex interplay of the nervous, endocrine, and immune systems, activating mechanisms such as the sympathetic–adreno–medullar (SAM) axis and the hypothalamic–pituitary–adrenal (HPA) axis [6]. While the stress response is typically adaptive, preparing the body to handle challenges, chronic or intense exposure to stressors can result in maladaptive outcomes, including depression, anxiety, and physical health impairments, such as cardiovascular disease [19,20,21,22]. Stress can take various forms, including acute stress, chronic stress, episodic acute stress, and traumatic stress, each with distinct physiological and psychological impacts. These forms of stress are often accompanied by psychosomatic symptoms affecting both the mind and body [1,23,24,25,26,27].

Quality of life (QoL) is a multidimensional concept that reflects an individual’s physical health, psychological state, social relationships, and level of independence within their environment and personal values [22,28,29,30,31,32,33,34,35,36]. It serves as a broad indicator of overall well-being, integrating both subjective and objective measures of health and satisfaction. Elevated stress levels have been shown to negatively influence QoL by impairing emotional well-being and resilience [1,37,38,39,40,41]. Chronic stress, in particular, disrupts physiological and psychological balance, reducing perceived QoL through mechanisms such as prolonged activation of the HPA axis [42,43]. Burnout, characterized by emotional exhaustion, depersonalization, and diminished personal accomplishment, further degrades QoL by undermining mental health and social functioning [1,44,45]. Additionally, disordered eating behaviors—often adopted as maladaptive coping mechanisms for stress—exacerbate the decline in QoL, creating a feedback loop that impacts physical and mental health [46,47,48]. Addressing these interconnected factors is critical for improving students’ QoL and academic performance.

In the university setting, stress often correlates with maladaptive coping mechanisms, including disordered eating behaviors [49,50]. Some students respond to stress by overeating, often favoring calorie-dense comfort foods, while others experience appetite suppression and engage in restrictive eating. Chronic stress is also a key contributor to burnout, a state of physical, emotional, and mental exhaustion that impairs academic performance, social interactions, and overall quality of life [51,52]. Burnout is characterized by three dimensions: emotional exhaustion, depersonalization, and reduced personal accomplishment [24,53]. Its emotional toll can disrupt self-care routines, including dietary practices, leading to behaviors such as skipping meals, binge eating, or turning to comfort foods, all of which influence eating attitudes. Given these effects, burnout may further exacerbate the negative impact of stress on students’ overall well-being and eating behaviors.

Research indicates that stress and burnout significantly influence eating attitudes, often contributing to patterns of disordered eating [33,54].

Elevated stress levels are associated with emotional eating or binge eating episodes, as students attempt to cope with negative emotions. Similarly, the mental exhaustion associated with burnout can impair decision-making regarding healthy eating, increasing the likelihood of maladaptive eating behaviors. Studies examining the relationship between eating attitudes, perceived stress, and burnout have reported significant correlations, with stress and burnout exacerbating unhealthy eating patterns [55,56]. This interplay often results in a vicious cycle, where disordered eating further deteriorates mental and physical well-being.

Given the prevalence of stress and burnout among students and their potential implications for eating behaviors, this study aims to explore these relationships in a sample of physical therapy students in Romania. By examining how perceived stress and burnout correlate with eating attitudes, this research seeks to provide insights into the psychological and behavioral factors contributing to students’ health and well-being. Specifically, this study investigates the influence of burnout dimensions and body mass index (BMI) as predictors of disordered eating patterns. Understanding these relationships is critical for developing targeted interventions to promote student well-being and mitigate the negative effects of stress and burnout on their eating behaviors and overall health.

## 2. Materials and Methods

This study included a total of 192 students enrolled at the Faculty of Midwifery and Nursing within the “Carol Davila” University of Medicine and Pharmacy in Bucharest. Participants were asked to complete three validated questionnaires: the Eating Attitudes Test (EAT-26) to assess eating behaviors [57,58,59] and the Perceived Stress Scale (PSS-14) to measure stress levels [60].

The Eating Attitudes Test (EAT-26) is a widely used standardized screening tool designed to identify potential disordered eating behaviors that may require professional attention. Developed by Garner et al. (1982), the test is not diagnostic but serves as an initial assessment to flag individuals who might benefit from further evaluation [57,58].

The test is divided into three sections:

- Demographic and Anthropometric Information: This includes questions about age, gender, height, current weight, ideal weight, and weight history.
- Attitudinal Statements: Participants respond to 26 statements about their thoughts, feelings, and behaviors related to food and eating. These are rated on a six-point Likert scale (Always, Usually, Often, Sometimes, Rarely, Never), addressing topics such as fear of weight gain, preoccupation with food, dieting behavior, and self-perception of body image.
- Behavioral Questions: This section assesses the frequency of specific behaviors in the past six months, such as binge eating, self-induced vomiting, use of laxatives or diuretics, excessive exercise, and significant weight loss.

The scoring and interpretation of the EAT-26 provide an understanding of specific areas of concern through its three subscales: Dieting, Bulimia and Food Preoccupation, and Oral Control.

- The Dieting Subscale focuses on behaviors and thoughts related to dieting and preoccupation with thinness (e.g., “I am terrified about being overweight.”, “I feel that food controls my life.”). A high score in this subscale indicates significant concerns about body weight and attempts to restrict food intake.
- The Bulimia and Food Preoccupation Subscale assesses binge eating behaviors and preoccupation with food (e.g., “I have gone on eating binges where I feel I may not be able to stop.”, “I vomit after I have eaten.”). High scores in this subscale suggest tendencies toward bulimia or obsessive thoughts about food.
- The Oral Control Subscale evaluates self-control around eating and the influence of social and familial expectations (e.g., “I avoid eating when I am hungry.”, “I feel that others would prefer if I ate more.”). A high score in this subscale reflects heightened concerns about eating behavior and external control.

The scores for all 26 items are summed to provide a total score. A total score of 20 or higher is considered a threshold for identifying individuals who may benefit from further evaluation for eating disorders. In addition to the total score, responses to behavioral questions about eating disorder behaviors (e.g., binge eating, vomiting, use of laxatives, etc.) provide further insight into the severity of disordered eating patterns.

In this study, the EAT-26 was used to analyze the relationships between stress, burnout, and eating attitudes among university students. The subscale scores provided insight into specific behavioral tendencies (e.g., dieting, bulimic behaviors, or oral control), while the total score highlighted students at higher risk of disordered eating. This comprehensive approach ensures a nuanced understanding of eating behaviors in the context of psychological stressors.

The Perceived Stress Scale (PSS-14), developed by Cohen, Kamarck, and Mermelstein in 1983, is a widely used psychological tool designed to measure the degree to which individuals perceive situations in their lives as stressful [60]. The PSS-14 is used in diverse populations and is applicable in assessing stress across various domains, including work, education, and health [61,62,63,64,65]. It has been validated for use in both clinical and non-clinical settings, with evidence supporting its reliability and validity across different cultures and age groups. The scale evaluates the extent to which respondents find their lives unpredictable, uncontrollable, and overwhelming, key components of the stress experience. It is particularly valued for its ability to assess subjective stress rather than objective stressors.

The PSS-14 consists of 14 items that assess perceived stress over the past month. Respondents rate each item on a 5-point Likert scale ranging from 0 (Never) to 4 (Very Often). The scale includes both positively and negatively worded items to reduce response bias:

- Positive items assess coping and control (e.g., “In the last month, how often have you felt confident about your ability to handle your personal problems?”).
- Negative items evaluate stress and helplessness (e.g., “In the last month, how often have you felt that things were going your way?”).

The total score is calculated by reversing the scores of the positively worded items and then summing all the responses.

The positive items need to be reverse scored (0 becomes 4, 1 becomes 3, 2 remains 2, 3 becomes 1, and 4 becomes 0). After reversing the positively worded items, all 14 item scores are summed to compute the total PSS-14 score. The total score ranges from 0 to 56, with higher scores indicating higher perceived stress levels.

- Scores ranging from 0 to 13 suggest low levels of perceived stress. Individuals in this range typically feel that their lives are manageable and experience minimal stress.
- Scores between 14 and 26 indicate moderate perceived stress, where individuals may feel occasionally overwhelmed or stressed but generally manage daily challenges.
- Scores above 27 suggest high levels of perceived stress, indicating significant feelings of unpredictability, uncontrollability, and emotional strain. Such individuals may benefit from stress-reducing interventions or professional support.

Higher scores indicate higher levels of perceived stress. The scale is not diagnostic but provides an important measure of stress perception that may contribute to understanding an individual’s psychological well-being.

In this study, the PSS-14 was employed to assess perceived stress levels among physical therapy students. This tool was selected due to its robustness and relevance in evaluating the subjective experience of stress, which plays a critical role in understanding the interplay between stress, burnout, and eating attitudes. The results from the PSS-14 offer valuable insights into the psychological state of the participants and their coping mechanisms.

In addition to the questionnaire responses, demographic and anthropometric data were collected from participants. These included age, sex, year of study, current weight, ideal weight, maximum weight ever recorded, minimum weight ever recorded, and height. This comprehensive dataset was used to examine the relationships between perceived stress, burnout, and eating attitudes, while also considering relevant personal and physical characteristics.

The questionnaires were distributed at the National Teaching Center for Children’s Neurorehabilitation “Dr. Nicolae Robanescu,” where students from the Faculty of Midwifery and Nursing at the University of Medicine and Pharmacy “Carol Davila” attend courses and practical training sessions. This study received approval from the institutional review board (No. 4160, dated 22 March 2024). Before proceeding with the survey, students were informed about this study’s purpose and provided their consent to participate.

### 2.1. Statistical Analysis

The statistical analysis was performed using Microsoft Excel (Microsoft Office Professional Plus 2021, Version 2501) and the Statistical Package for the Social Sciences IBM SPSS Statistics, Version 26. Data normality was assessed using the Shapiro–Wilk test to determine the appropriate statistical tests for analysis.

Spearman’s correlation was used to identify relationships between variables that were not normally distributed.

ANOVA (Analysis of Variance) was applied to compare means across multiple groups for normally distributed data.

The Kruskal–Wallis test, a non-parametric alternative to ANOVA, was used for comparing medians between groups when data did not meet normality assumptions.

Significance levels were set at *p* < 0.05, and all tests were two-tailed. These methods ensured a robust analysis of the relationships between variables in this study.

### 2.2. General Objective and Hypothesis of This Study

The general objective of this study was to examine the relationships between perceived stress, eating behaviors, and BMI among university students, focusing on the influence of burnout dimensions and BMI as predictors of disordered eating patterns.

This study tested the following hypotheses:

General hypothesis:

- Perceived stress, BMI, and weight-related perceptions are significant predictors of disordered eating behaviors among university students.

Specific hypotheses:

- Stress and Eating Behavior: Higher perceived stress is associated with disordered eating behavior.
- BMI and Eating Behavior: Individuals with a BMI outside the normal range have a greater risk of disordered eating.
- Gender Differences: Women have a higher risk of disordered eating and greater perceived stress than men.
- Weight Perception and Eating Behavior: Students with a perceived gap between current and ideal weight have higher EAT-26 scores.
- Weight Fluctuations: Students with significant weight fluctuations are more predisposed to disordered eating.
- Oral Control and BMI: Oral control predicts BMI.
- Age and Stress: Older students experience higher levels of perceived stress.

## 3. Results

**Figure 1:**
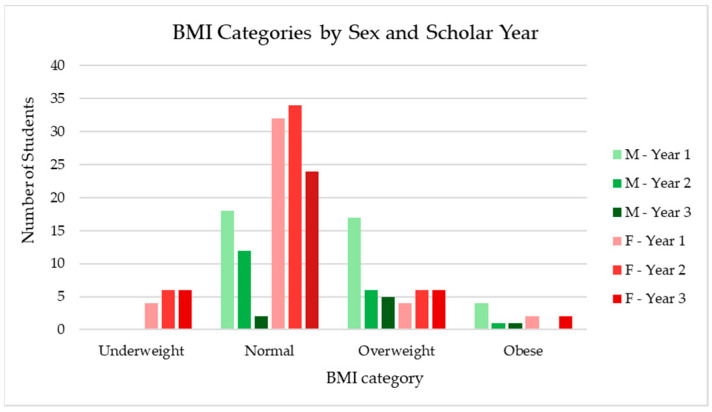
Distribution of respondents by gender, BMI category, and academic year.

The demographic characteristics of the respondents are summarized in Table 1 and illustrated in Figure 1. Males accounted for 34.37% of the respondents, while females represented 65.63%.

In terms of academic year distribution, 42.17% of the respondents were in their first year, 33.87% in their second year, and 23.96% in their final year of university.

BMI was calculated based on self-reported height (in cm) and weight (in kg). BMI categories were defined as follows: Underweight: BMI < 18.5, Normal weight: BMI 18.5–24.9, Overweight: BMI 25–29.9, and Obese: BMI > 30 [66].

The distribution of respondents by BMI category was as follows: 8.3% were underweight, 63.6% had a normal weight, 22.9% were overweight, and 5.2% were obese. The mean BMI of the respondents was 23.214 (SD = 4.139), placing the average in the normal weight range.

The findings indicate that male students had a higher mean BMI (25.59) compared to female students (21.96).

**Figure 2:**
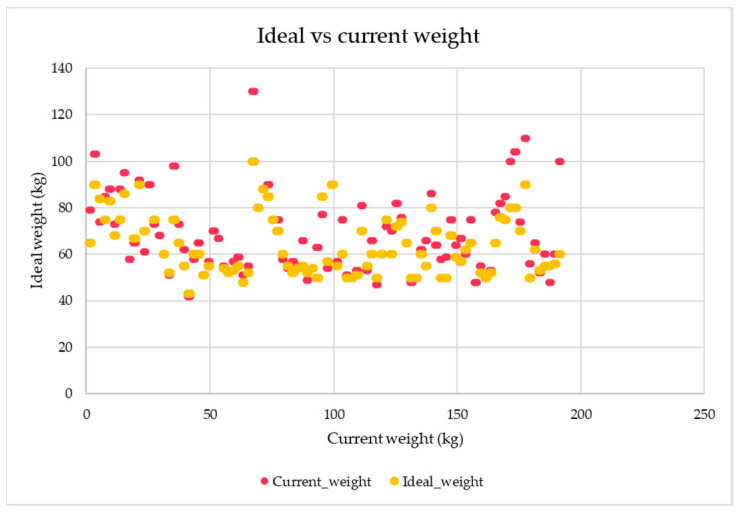
Relationship between current weight and ideal weight among respondents.

Figure 2 illustrates the relationship between the respondents’ current weight and their self-reported ideal weight. The scatterplot highlights individual differences and trends, providing insight into how closely respondents perceived ideal weights align with their actual weights.

The relationship between the respondents’ current and ideal weight is presented with specific data. Among male participants, the average current and ideal weights were as follows:

- Year 1: 84.48 ± 15.63 kg vs. 79.91 ± 8.995 kg (a 5.41% difference);
- Year 2: 79.58 ± 10.259 kg vs. 75.68 ± 9.464 kg (a 4.90% difference);
- Year 3: 94.88 ± 12.8 kg vs. 78.88 ± 5.74 kg (a 16.86% difference).

For female participants, the average current and ideal weights were as follows:

- Year 1: 60.36 ± 12.445 kg vs. 56.17 ± 7.284 kg (a 6.94% difference);
- Year 2: 57.57 ± 8.148 kg vs. 55 ± 4.899 kg (a 4.46% difference);
- Year 3: 63.25 ± 12.357 kg vs. 57.50 ± 6.372 kg (a 9.09% difference).

**Figure 3:**
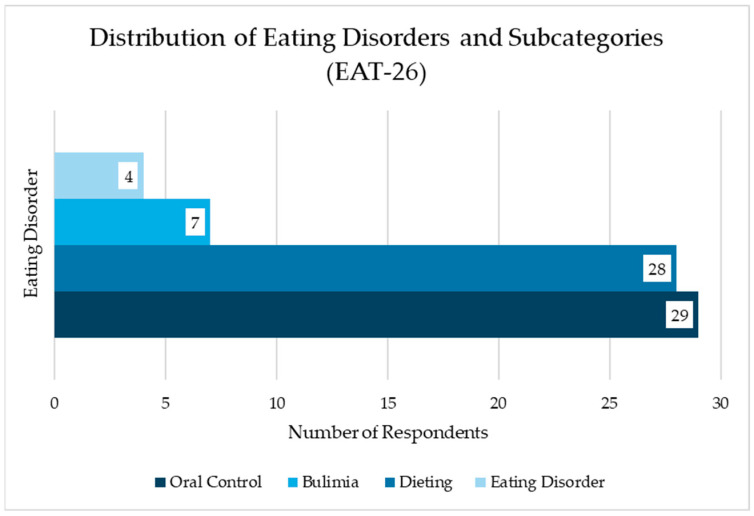
Distribution of eating disorders and subcategories identified by EAT-26.

From the *EAT-26 test*, it was found that 29 out of the 192 respondents exhibit disordered eating behaviors, as shown in Figure 3. Among these 29 individuals, 28 display behaviors specific to dieting, 7 show tendencies toward bulimia (including the one not categorized under dieting), and 4 exhibit oral control disorders (also including the one not categorized under dieting).

Of the 29 respondents with disordered eating behaviors, 3 are underweight, 17 have a normal weight, 6 are overweight, and 3 are obese.

Among the 29 respondents with disordered eating behaviors:

- A total of 7 individuals (4 with normal weight, 1 overweight, and 2 obese) experienced binge eating more than once a week.
- A total of 3 individuals (1 overweight and 2 obese) reported self-induced vomiting multiple times per week.
- A total of 4 individuals (2 with normal weight and 2 obese) used laxatives or diuretics multiple times per week to control their weight.
- A total of 4 individuals (3 with normal weight and 1 overweight) engaged in physical exercise for more than 60 min a day at least once a week.
- A total of 16 individuals (10 with normal weight, 3 overweight, and 3 obese) engaged in physical exercise for more than 60 min a day multiple times per week to reduce or control their weight.
- A total of 9 individuals (2 underweight, 4 with normal weight, and 3 overweight) reported losing 9 kg or more in the past six months.

In the assessment of gender differences in eating behaviors, male students had higher scores on the Dieting subscale (10.61 vs. 8.02), whereas female students scored slightly higher on the Bulimia and Food Preoccupation (1.81 vs. 1.67) and Oral Control (3.67 vs. 3.36) subscales.

Table 1 presents the descriptive statistics for the Perceived Stress Scale (PSS-14) and the Eating Attitudes Test (EAT-26) subscales, including mean scores, standard deviations, minimum and maximum values, and 95% confidence intervals. These results provide an overview of perceived stress and eating behaviors within the study sample, offering a general perspective on the distribution of these variables.

The *perceived stress levels* among respondents are summarized in Table 1. The majority of participants reported moderate or high stress levels, with 50% experiencing moderate stress and 42% reporting high stress. Only a small proportion (8%) indicated low stress levels. These findings highlight the prevalence of stress among the study population, emphasizing the need to address factors contributing to elevated stress levels in university students.

Among those reporting high stress, females represented 81.48%, compared to 62.3% among those with moderate stress and 37.5% among those with low stress. These findings indicate that females tend to experience higher levels of perceived stress, highlighting the need for gender-specific stress management interventions within the student population. In the assessment of gender differences in perceived stress levels, female students had slightly higher mean scores on the PSS-14 scale (25.52 vs. 24.33 for male students).

The correlation analysis reveals the following relationships:

1. *The relationship between stress and eating behavior*

*Higher perceived stress is associated with increased dieting, bulimia, and food preoccupation, as shown by PSS-14 correlations:*

  - A moderate positive correlation with Dieting (r = 0.3, *p* < 0.001), indicating that higher perceived stress is associated with increased dieting behaviors.
  - A moderate positive correlation with Bulimia and Food Preoccupation (r = 0.329, *p* < 0.001), suggesting that higher perceived stress is linked to higher tendencies of bulimia and food preoccupation.
  - A moderate positive correlation with Oral Control (r = 0.302, *p* < 0.001), showing that higher stress levels are related to more pronounced oral control behaviors.

Dieting correlations:

  - A positive correlation with Bulimia and Food Preoccupation (r = 0.394, *p* < 0.001), indicating that individuals engaging in dieting behaviors are more likely to exhibit tendencies toward bulimia and food preoccupation.
  - A weak positive correlation with Oral Control (r = 0.161, *p* = 0.025), suggesting a slight association between dieting and oral control behaviors.

Bulimia and Food Preoccupation correlations:

  - A positive correlation with Oral Control (r = 0.325, *p* < 0.001), showing that individuals with higher tendencies toward bulimia and food preoccupation are more likely to exhibit oral control behaviors.

2. *The relation between BMI and eating behavior*
  - Oral Control showed no significant differences across BMI groups (F(3, 188) = 2.517, *p* = 0.060). Although underweight participants exhibited higher mean scores (M = 4.50, SD = 3.23) compared to overweight (M = 2.61, SD = 2.97) and obese participants (M = 3.20, SD = 3.62), post hoc analyses (Tukey HSD and Bonferroni) revealed no statistically significant pairwise differences (*p* > 0.05);
  - For the Dieting component, no significant effect of BMI was found (F(3, 188) = 0.373, *p* = 0.773). The mean scores were similar across all BMI groups, with obese participants having the highest mean score (M = 11.00, SD = 9.80);
  - In the Bulimia and Food Preoccupation component, there was no significant difference between BMI categories (F(3, 188) = 2.149, *p* = 0.096). However, the post hoc analysis indicated a near-significant difference between obese and overweight participants (*p* = 0.058), with obese participants displaying higher levels of bulimic tendencies (M = 3.50, SD = 3.89) compared to other BMI groups.

3. *Gender differences in eating behavior and perceived stress*

Disordered eating behavior by gender:

  - Men (M = 15.64, SD = 9.79, *n* = 66) had a higher mean EAT-26 score compared to women (M = 13.51, SD = 9.47, *n* = 126). However, the relatively small sample size and the uneven distribution of subgroups, such as gender and BMI categories, limit the robustness of the results.

Perceived stress by gender:

  - Men (M = 24.33, SD = 7.22, *n* = 66) had slightly lower mean stress scores compared to women (M = 25.52, SD = 8.02, *n* = 126), although the difference in mean PSS-14 scores was not statistically significant.

The relationship between disordered eating behaviors and perceived stress levels:

  - The Pearson Correlation (r = 0.468, *p* < 0.001) indicates a moderate, positive correlation between EAT-26 and PSS-14 scores. Higher levels of perceived stress are associated with more pronounced disordered eating behaviors.
  - The Spearman’s Rho (r_s_ = 0.439, *p* < 0.001) analysis confirms the relationship observed in the Pearson correlation. The result suggests that even if the assumptions of normality are violated, the positive association between stress and disordered eating remains robust.

Logistic regression analysis showed that sex and perceived stress (PSS-14) significantly improved the prediction of disordered eating risk (χ^2^(2) = 35.220, *p* < 0.001), explaining 25.3% of the variance (Nagelkerke R² = 0.253). The model had an overall accuracy of 80.7%, correctly identifying 94.6% of participants not at risk and 35.6% of those at risk. For each 1-point increase in perceived stress, the odds of disordered eating increased by 16.1% (B = 0.150, *p* < 0.001, Exp(B) = 1.161). Men were 2.16 times more likely to display disordered eating behaviors compared to women, with this result approaching significance (B = 0.769, *p* = 0.051, Exp(B) = 2.158).

4. *Perceived weight discrepancy and eating behavior*
  - The Pearson Correlation (r = 0.030, *p* = 0.691) between the weight gap (difference between current and ideal weight) and EAT-26 scores is positive but very weak and not statistically significant (*p* > 0.05).

5. *Weight fluctuation history*

A linear regression was conducted to evaluate whether weight fluctuation (difference between maximum and minimum reported weight) predicts disordered eating behaviors as measured by the EAT-26 score.

  - The correlation between weight fluctuation and EAT-26 scores was weak (R = 0.095), with weight fluctuation explaining 0.9% of the variance in EAT-26 scores (R² = 0.009).
  - For each 1 kg increase in weight fluctuation, the EAT-26 score is predicted to increase by 0.107 points. However, this relationship is not statistically significant (*p* > 0.05).

6. *Oral control predicts BMI*

The regression analysis showed a moderate correlation between the predictors (Dieting, Bulimia and Food Preoccupation, Oral Control) and BMI (R = 0.414). The model explained 17.2% of the variance in BMI (R² = 0.172), although the adjusted R² (0.116) indicated a lower proportion after accounting for the number of predictors. The standard error of the estimate was 3.90, reflecting the average deviation of predicted BMI values from observed values. The model was statistically significant (F(6,89) = 3.072, *p* = 0.009), suggesting the predictors had a meaningful impact on BMI.

The regression coefficients indicated the following:

  - Dieting (B = 0.072, Beta = 0.097, *p* = 0.356) was not a significant predictor of BMI.
  - Bulimia and Food Preoccupation (B = 0.093, Beta = 0.038, *p* = 0.715) did not significantly predict BMI.
  - Oral Control (B = -0.564, Beta = -0.379, *p* < 0.001) was a significant negative predictor of BMI, with each unit increase in oral control associated with a 0.564 unit decrease in BMI.

7. *Younger students experience higher levels of perceived stress*

There is a significant positive correlation between perceived stress and age, indicating that older students report higher levels of perceived stress.

The correlation analysis showed a positive relationship between perceived stress (PSS-14) and age (r = 0.285, *p* < 0.001), indicating that stress levels tend to increase slightly with age. This correlation was statistically significant at the 0.01 level.

## 4. Discussion

Our study highlights a significant interplay between stress and eating behaviors among physical therapy students. These stress levels are correlated with disordered eating behaviors, particularly dieting and bulimia/food preoccupation. Emotional eating and maladaptive dietary patterns likely serve as coping mechanisms for stress, reflecting previous research findings.

### 4.1. Stress and Eating Behavior: Higher Perceived Stress Is Associated with Disordered Eating Behavior

The analysis revealed significant positive correlations between perceived stress levels and disordered eating behaviors, with varying degrees of association across the EAT-26 subscales. Specifically, perceived stress showed a moderate correlation with dieting behaviors, suggesting that students experiencing higher stress levels are more likely to engage in restrictive eating patterns as a potential coping mechanism, as highlighted in recent literature [67,68]. Furthermore, the strongest correlation was observed between perceived stress and the bulimia/food preoccupation subscale, indicating that elevated stress may contribute to impulsive eating episodes, emotional eating, or an increased preoccupation with food. This aligns with the existing literature that highlights stress as a key trigger for binge eating and loss of control over food intake [69,70]. A positive correlation was also found between stress and the oral control subscale, reflecting a tendency among stressed students to exert stricter control over eating habits, possibly as a compensatory strategy to regain a sense of control in the face of psychological distress, as highlighted in the literature [71].

Our findings underscore the complex interplay between stress and disordered eating behaviors, where students may oscillate between restrictive dieting, heightened food preoccupation, and attempts at rigid control over food intake. This pattern highlights the need for tailored stress management interventions that also address maladaptive coping strategies related to eating behaviors. By mitigating stress, such interventions could potentially reduce the risk of developing disordered eating patterns among university students.

### 4.2. BMI and Eating Behavior: Individuals with a BMI Outside the Normal Range Have a Greater Risk of Disordered Eating

The hypothesis stating that individuals with a BMI outside the normal range have a greater risk of disordered eating was not supported by the data. Correlational analyses revealed no significant associations between BMI and any of the EAT-26 subscales (Dieting, Bulimia/Food Preoccupation, and Oral Control). These findings suggest that, within this sample, BMI does not appear to be a predictor of disordered eating behaviors. This result contrasts with previous research that has often identified higher BMI as a risk factor for disordered eating but may reflect the specific characteristics of the study population, such as their academic background in health sciences, which could influence their attitudes toward weight and eating behaviors [71].

### 4.3. Gender Differences: Women Have a Higher Risk of Disordered Eating and Greater Perceived Stress than Men

Among students experiencing high levels of perceived stress, 81.48% were female, indicating a marked gender disparity in stress responses. Similarly, 62.3% of those with moderate stress were female, compared to 37.5% of those reporting low stress. These findings suggest that female students are more prone to higher stress levels, potentially reflecting gender-specific vulnerabilities to academic and social pressures. This aligns with prior research emphasizing the need for tailored stress management programs for female students [20,72]. Despite these gender differences in stress, no significant association was found between BMI and the components of EAT-26. While trends suggested higher Oral Control and Bulimic tendencies among underweight and obese participants, the differences were not statistically significant.

Regarding gender differences in eating behavior, the results indicated no significant difference in disordered eating patterns between men and women based on EAT-26 scores. Although men had slightly higher mean scores, the difference was not statistically significant.

### 4.4. Weight Perception and Eating Behavior: Students with a Perceived Gap Between Current and Ideal Weight Have Higher EAT-26 Scores

No significant relationship was found between weight fluctuation and disordered eating behaviors. The hypothesis that students with larger discrepancies between their actual and ideal weight would score higher on the EAT-26 was not supported. This suggests that other factors, such as stress, psychological well-being, or societal pressures, may play a more critical role in disordered eating behaviors.

### 4.5. Weight Fluctuations: Students with Significant Weight Fluctuations Are More Predisposed to Disordered Eating

The hypothesis that significant weight fluctuations increase the risk of disordered eating behaviors was not supported by the findings of this study. Although the analysis suggested a slight tendency for students with greater weight fluctuations to exhibit higher disordered eating scores, the relationship was weak and not statistically significant. This indicates that, within this sample, weight fluctuation alone does not serve as a meaningful predictor of disordered eating behaviors. These results contrast with studies that have identified weight variability as a potential risk factor for disordered eating, particularly in populations with heightened body image concerns or those engaged in frequent dieting [73,74]. The current findings may reflect the characteristics of the study population: physical therapy students who, given their academic background in health sciences, may adopt more balanced approaches to weight management, thus reducing the likelihood of extreme dietary behaviors. Additionally, the absence of a significant association could be influenced by the self-reported nature of weight data, which may introduce recall bias, or by the homogeneity of the sample in terms of age, lifestyle, and academic context. It is also possible that other psychological factors, such as body dissatisfaction or stress-coping strategies, mediate the link between weight fluctuations and disordered eating but were not examined in this study.

Our findings highlight the complexity of disordered eating behaviors and suggest that weight fluctuation, in isolation, may not be a strong predictor, underscoring the need to consider a broader range of influencing factors when assessing risk.

### 4.6. Oral Control and BMI: Oral Control Predicts BMI

The hypothesis that oral control predicts BMI was partially supported by the findings of this study. The overall regression model demonstrated that the combination of dieting behaviors, bulimia and food preoccupation, and oral control had a meaningful impact on BMI. Examining the individual predictors, we found that only oral control emerged as a significant factor influencing BMI. The relationship between oral control and BMI was negative, suggesting that higher levels of oral control are associated with lower BMI values. This finding aligns with the existing literature that highlights the role of restrictive eating patterns in weight management [75]. Individuals who exhibit greater self-regulation over their food intake—often characterized by behaviors such as portion control, delayed gratification, and heightened awareness of eating habits—tend to maintain lower body weights. In contrast, less emphasis on oral control may lead to more impulsive eating behaviors, potentially contributing to higher BMI.

On the other hand, dieting and bulimia/food preoccupation did not significantly predict BMI in this sample. This could be attributed to the complex nature of these behaviors, where the intention to diet does not always translate into effective weight management, and bulimic tendencies may result in weight fluctuations rather than consistent changes in BMI. Additionally, the lack of significant associations may reflect the characteristics of the study population: physical therapy students, who may be more informed about health behaviors, leading to more controlled eating practices despite occasional disordered eating patterns.

These findings underscore the importance of considering the nuanced roles that specific eating behaviors play in influencing BMI. While general dieting efforts may not have a direct impact on body weight, the degree of conscious control over eating appears to be a more influential factor. This insight highlights the potential benefits of interventions that promote mindful eating and self-regulation strategies, particularly in populations concerned with health and body image.

### 4.7. Age and Stress: Older Students Experience Higher Levels of Perceived Stress

Perceived stress levels were predominantly moderate to high, with older students reporting elevated stress compared to their younger peers.

However, a statistically significant positive correlation was found between perceived stress and disordered eating behaviors. This suggests that higher stress levels increase the risk of disordered eating patterns, supporting the hypothesis that stress may contribute to the development or exacerbation of such behaviors.

The logistic regression model confirmed that perceived stress (PSS14) is a significant predictor of disordered eating risk, with higher stress levels associated with greater odds of disordered eating. Although sex had a marginally significant effect, the data suggested that men may have a higher risk of disordered eating compared to women.

Students in medical universities frequently experience a combination of psychological and behavioral symptoms, with stress, burnout, and eating disorders being prevalent [49,52,76,77,78]. Universities should prioritize interventions that integrate mental health support, stress reduction techniques, and nutritional education tailored to students’ academic trajectories [79].

## 5. Limitations

This study has several limitations that should be considered when interpreting the findings. Firstly, the sample was homogeneous, consisting exclusively of physical therapy students from “Carol Davila” University of Medicine and Pharmacy, which limits the generalizability of the results to other academic disciplines and socioeconomic backgrounds. Additionally, the lack of cultural diversity further restricts the external validity. Future studies should include more diverse populations to enhance generalizability.

The relatively small sample size, despite including all eligible students, limited the power of subgroup analyses, such as gender differences. Expanding the sample across multiple institutions could improve the robustness of future analyses.

This study found no significant associations between BMI and disordered eating behaviors, which may reflect the specific characteristics of the sample or the complexity of these behaviors. Future research should use non-parametric tests, larger samples, and explore psychological mediators like body dissatisfaction or coping strategies. Similarly, while weight fluctuation showed a slight association with disordered eating, the relationship was weak and not statistically significant, potentially due to self-reported data and sample homogeneity. Longitudinal studies could offer deeper insights into how weight changes over time relate to eating behaviors.

Although oral control emerged as a significant negative predictor of BMI, this study did not account for potential moderating factors such as perfectionism or stress-coping strategies. Future research should explore these variables and use longitudinal designs to assess stability over time.

Finally, the cross-sectional design limits causal inferences, preventing conclusions about the directionality of relationships between stress, BMI, and disordered eating. Longitudinal studies are recommended to better understand these dynamics.

## 6. Conclusions

This study highlights the complex relationship between perceived stress and disordered eating behaviors among Romanian physical therapy students. Higher stress levels correlated with increased tendencies for disordered eating, particularly in dieting and bulimia/food preoccupation, supporting the hypothesis that stress may trigger or exacerbate maladaptive eating patterns.

Female students reported higher levels of perceived stress compared to their male counterparts. However, disordered eating patterns did not differ significantly between genders, despite men showing slightly higher mean EAT-26 scores.

No significant relationship was found between BMI and disordered eating behaviors. While trends suggested that underweight and obese students might exhibit greater bulimic tendencies and oral control, these differences were not statistically significant. Additionally, weight fluctuation was not correlated with EAT-26 scores, suggesting that other psychological and environmental factors may play a larger role in disordered eating.

A key finding was the positive correlation between perceived stress and disordered eating, emphasizing stress as a critical factor influencing students’ eating patterns. Logistic regression analysis reinforced this, identifying perceived stress as a significant predictor of disordered eating risk.

These results underline the importance of addressing stress in the university environment, especially for female students, who may be more vulnerable to its effects. Universities should prioritize comprehensive interventions that incorporate mental health support, stress reduction programs, and nutritional education to mitigate the risk of disordered eating and enhance overall student well-being.

Future research should explore additional psychological and social factors contributing to disordered eating, with an emphasis on longitudinal studies and larger, more diverse student populations. Addressing stress as a modifiable risk factor for disordered eating could improve both student health and academic performance. This study’s limitations, including its small sample size and focus on a single university, should be acknowledged and addressed in subsequent research.

## Figures and Tables

**Table 1:** Summary of PSS-14 and EAT-26 scores with confidence intervals.

Scores	N	Minimum	Maximum	Mean	Std. Deviation	95% Confidence Interval for Mean	
						Lower Bound	Upper Bound
PSS14	192	7	42	25.11	7.752	24.01	26.21
Dieting	192	0	28	8.91	6.829	7.94	9.88
Bulimia and Food Preoccupation	192	0	11	1.76	2.431	1.41	2.11
Oral Control	192	0	11	3.57	2.924	3.15	3.98

## Data Availability

All data are contained in the manuscript.
